# Molecular Phylogeny of *Trifolium* L. Section *Trifolium* with Reference to Chromosome Number and Subsections Delimitation

**DOI:** 10.3390/plants10101985

**Published:** 2021-09-23

**Authors:** Hanan I. Sayed Ahmed, Abdelfattah Badr, Hanaa H. El-Shazly, Linda Watson, Ahmed S. Fuoad, Faten Y. Ellmouni

**Affiliations:** 1 Botany and Microbiology Department, Faculty of Science, Tanta University, Tanta 31527, Egypt; hanan.ahmed@science.tanta.edu.eg; 2 Botany and Microbiology Department, Faculty of Science, Helwan University, Cairo 117900, Egypt; abadr@science.helwan.edu.eg; 3 Department of Biological Sciences and Geology, Faculty of Education, Ain Shams University, Cairo 11341, Egypt; hhelshazly@yahoo.com; 4 Department of Plant Biology, Ecology, and Evolution, Oklahoma State University, Stillwater, OK 74078-3013, USA; linda.watson10@okstate.edu; 5 Botany and Microbiology Department, Faculty of Science, Cairo University, Giza 12613, Egypt; 6 Botany Department, Faculty of Science, Fayoum University, Fayoum 63514, Egypt; fyl00@fayoum.edu.eg

**Keywords:** *Trifolium*, phylogeny, ITS sequence, section *Trifolium*, chromosome number

## Abstract

The genus *Trifolium* is one of the largest genera of the legume family Fabaceae with ca. 255 species. The genus is divided into eight sections; the section *Trifolium* is a major section of the genus, comprising 73 species mainly distributed in the Mediterranean region. We used nuclear ribosomal DNA internal transcribed spacer (ITS) and morphological variation to reconsider the delimitation and phylogenetic relationships of species in the section *Trifolium* with reference to chromosomal variations. Bayesian analysis of ITS data delimited the species as three clades based on the analysis of ITS sequence and informative indels in combination with morphological variation. The phylogeny of the species by different analyses methods does not support their current delimitation in 17 subsections. The basic chromosome number *x* = 8 is the number for the genus *Trifolium*, from which *x* = 7, 6 and 5 were derived through successive aneuploidy events. With reference to the distribution of these numbers in the species of the section *Trifolium*, species in clade III and clade II are more evolved than species in clade I.

## 1. Introduction

The clover genus *Trifolium* L. is one of the most important genera of the family Fabaceae. It comprises approximately 255 herbaceous, perennial and annual species distributed in different geographic regions, particularly in the Mediterranean region, East Europe, Eurasia, the highlands of eastern Africa and western North America [1,2,3]. The economic importance of the genus is demonstrated by the wide growth of at least 16 species as livestock forage and green manure crops [2], and by the capacity of over 125 species to fix nitrogen through root nodulation by the bacterium *Rhizobium leguminosarum* biovar. Trifolii [4]. Fertile interspecific hybrids are difficult to achieve in *Trifolium* [5], and only succeed between closely related species [6,7,8,9]. Williams et al. [10] supported the hypothesis that a diploid alpine species (*T. pallescens*) mated with a diploid coastal species (*T. occidentale*) to produce tetraploid *T. repens.* This has spurred interest in the evaluation of the agronomic potential of locally utilized and currently uncultivated species as a potential genetic resource for crops [11].

Most contemporary classifications treat *Trifolium* sensu lato as one large genus of eight sections [12,13,14], of which six are restricted to the Old World (*Chronosemium*, *Mistyllus*, *Paramesus*, *Trichocephalum*, *Trifolium*, *Vesicaria*), one to the New World (*Involucrarium*) and one occurs in both hemispheres (*Lotoidea*). The later section comprises over 95 species and has traditionally been considered ancestral to all other sections due to its worldwide distribution and morphological heterogeneity [13,14].

The *Trifolium* section has a native distribution throughout continental Europe, extending to Eurasia and south to North Africa. Species of this section show diverse specializations in seed dispersal mechanisms [14]. The section is considered one of the most derived sections in the genus on the basis of several features including a relatively large proportion of annual species, predominantly diploid species with a tendency towards descending aneuploidy [15,16], highly specialized seed dispersal mechanisms, a reduction to a single-seeded pod [14] and heterogeneity in seed proteins [17].

The available literature about the subsectional delimation of the section *Trifolium* is contradictory. Zohary and Heller [14] proposed 17 subsections of one to ten species each. However, this sub-sectional delimitation has been found incongruent, with variation in seed protein electrophoretic patterns and the interspecific relationships as judged by the ability of species to cross [6,7,8].

Molecular phylogeny of Old World *Trifolium* species showed the *Trifolium* section to be polyphyletic, and the placement of the Mediterranean section *Chronosemium* (21 species) remained unresolved with conflicting hypotheses such as being derived from within the section *Trifolium* in a nrDNA phylogeny vs. sister to the entire genus in a cpDNA phylogeny [16]. Ellison et al. [1] provided a comprehensive systematic revision of the genus *Trifolium* using parsimony and Bayesian phylogenetic analyses based on nuclear ribosomal DNA internal transcribed spacer and chloroplast *trn*L intron sequences. The authors proposed dividing the genus into two subgenera, subg. *Chronosemium* and subg. *Trifolium*; the latter was divided into eight sections which are not in agreement with the sub-sectional delimitations of Zohary and Heller [14]. The ancestral life history was also assumed to be annual in subg. *Chronosemium* and indefinite in subg. *Trifolium*. However, transitions between the annual and perennial habit are common [18].

Comparative seed characteristics of *Trifolium* species [19] and comparative analysis of qualitative anatomical traits [20] indicated some diagnostic characters for the identification of *Trifolium* sections, which contradict the sub-sectional delimitation based on molecular phylogeny. Moreover, the sub-sectional delimitation of species in the section *Trifolium* is not congruent with the number of clades resolved by Watson et al. [16] and by Ellison et al. [1]. The use of non-morphological types of information certainly adds to the informational content for plant classifications. However, they should be integrated with (but not substitute) morphological data [21]. Character states were also reconstructed from chromosomal variations using 2n = 16 as the ancestral chromosome number in *Trifolium* and an inferred 19 instances of aneuploidy and 22 of polyploidy in the genus [17,22]. All chromosome numbers reported for the whole genus are based on the basic numbers of *x* = 8, 7, 6 and 5 [14,15,23,24,25,26]. These studies have indicated the changes in chromosome number as a major player in the evolution of *Trifolium*.

The internal transcribed spacer sequence (ITS) of the ribosomal DNA has been demonstrated to be an accurate source of information for examining molecular phylogeny in many plant families including the Fabaceae [27,28]. Indels in the ITS sequences are consistently present in most alternative alignments and are more reliable for phylogenetic analysis [29]. The ITS indels have been successfully applied in the genus *Trifolium* at the sectional level [1,16] and at the species level [30]. Jreisat and Laten [31] recommended ITS for accurate identification and labeling of plant germplasm and for basic genetic and evolutionary studies to avoid mislabeled or misidentified germplasm collections.

Therefore, the objective of the present study is to construct a phylogenetic relationship of species in the *Trifolium* sect. *Trifolium* aims to reconsider the sub-sectional delimitation of species in this section, based on a comprehensive sampling of species, ITS analysis and variation in selected morphological traits in the light of variations in chromosome numbers of the examined species.

## 2. Results

**Figure 1:**
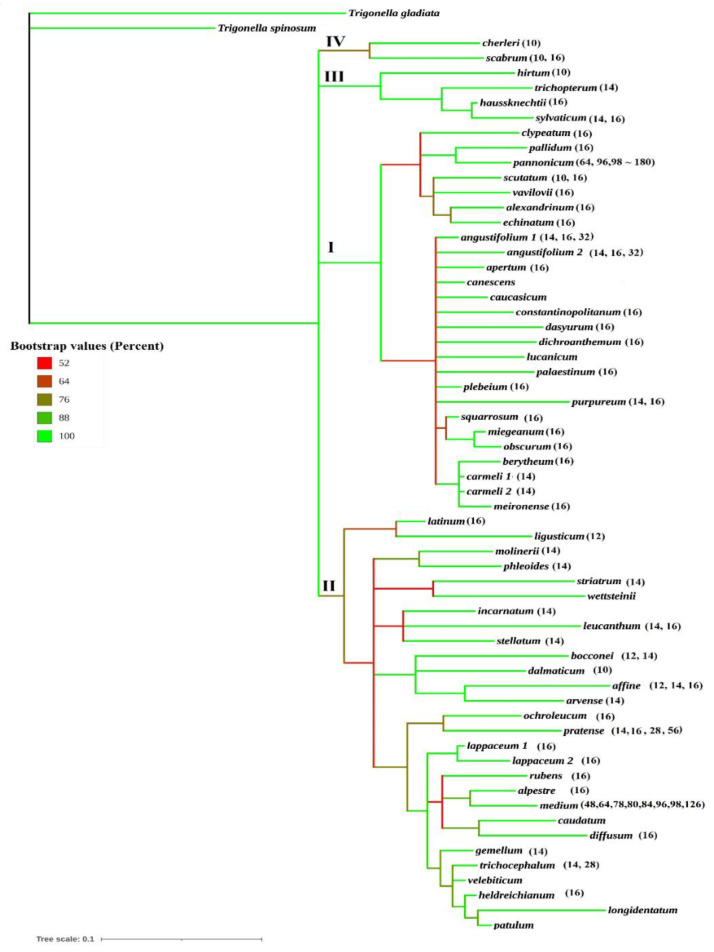
Phylogenetic tree expressing the classification of the species of the section *Trifolium* based on the ITS sequence and informative indels using MrBayes software and applying the Interactive Tree of Life (iTOL) tools. The colors of lines indicate the bootstrap value (percentage) for the separation of clades as expressed in the tree. The delimitation of the species as four clades is supported by a bootstrap value.

Figure 1 is a phylogenetic tree expressing the classification of the species of the section *Trifolium* based on the ITS sequence and informative indels, using MrBayes 3.2 [32] applying Interactive Tree of Life (iTOL) tools [33]. This Figure illustrates that all species of the section *Trifolium* are clearly isolated from the two outgroups of *Trigonella*, i.e., *Trigonella gladiata* and *Trigonella spinosum*. The *Trifolium* species are divided into two major clades: clade I comprises 26 species and clade II 28 species, in addition to two minor clades, clade III of 4 species and clade IV of 2 species. Clade I with a strongly supported bootstrap value of 100 is differentiated into two subclades, one of 7 species and the other of 19 species. The seven species of the first subclade are *T. alexandrinum*, *T. echinatum*, *T. scutatum*, *T. vavilovii*, *T. clypeatum*, *T. pallidum* and *T.* *pannonicum*. In the second subclade, *T. berytheum* is grouped with the two samples of *T. carmeli* 1, 2, and *T. meironense*, while *T. squarrosum* is clustered with *T. miegeanum* and *T. obscurum*. The other 12 species in this subclade are differentiated as single branches (Figure 1). In clade II, *T. latinum* and *T. ligusticum* are separated as a small subclade and the other 26 species are differentiated into one large subclade of 15 species and four small subclades, one of four species (*T. affine*, *T. arvense*, *T. bocconei* and *T. dalmaticum*), one of three species (*T. incarnatum*, *T. leucanthum*, *T. stellatum*) and two small subclades of two species each, i.e., *T. molinerii* grouped with *T. phleoides* and *T. striatum* with *T. wettsteinii* (Figure 1). On the other hand, clade III contained *T.* *haussknechtii*, *T. sylvaticum*, *T. trichopterum* and *T. hirtum*, while *T. cherleri* and *T. scabrum* were clustered in clade IV. The colors of the tree branches indicate the bootstrap values representing the percentage for the separation of clades and species. These values clearly show that the delimitation of the species in the section *Trifolium* as four clades. The number between parenthesis after the species branches are the recorded chromosome numbers for the species. All species in clade I have a basic chromosome number of *x* = 8 except *T. purpureum* and all are diploid with 2n = 16 except *T. pannonicum* (2n = 64, 96, 98~180). On the other hand, chromosome numbers based on *x* = 5, *x* = 6, and *x* = 7 have been reported in fourteen species in clade II and all the six species in clades III and IV.

**Figure 2:**
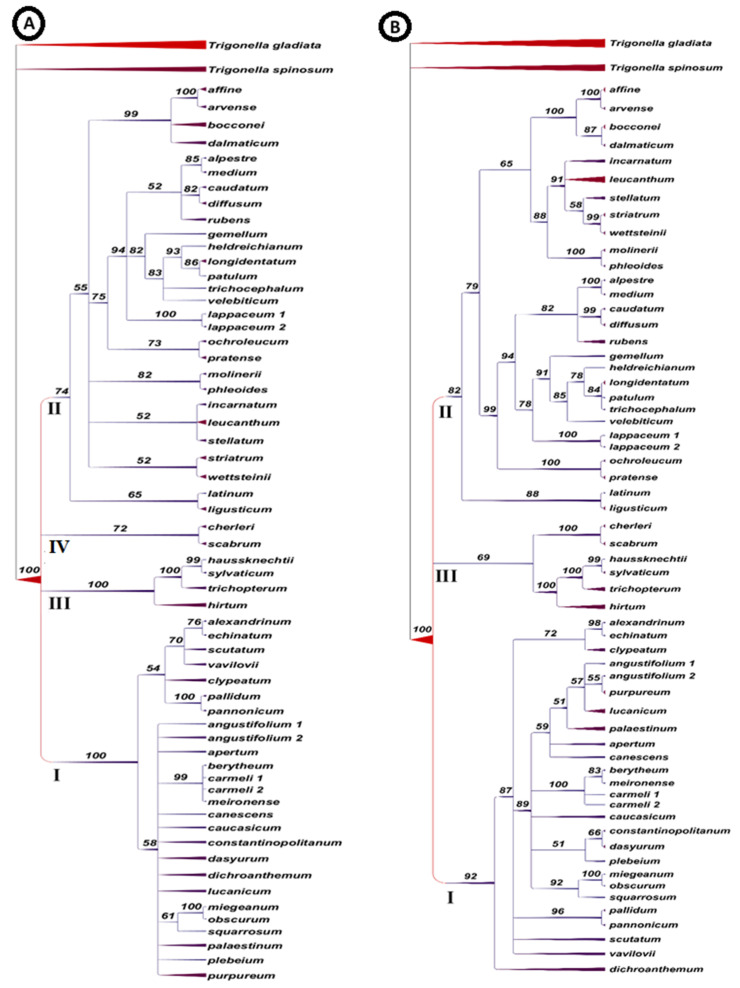
Two phylogenetic trees of the species in the section *Trifolium* constructed using MrBayes using TreeGraph 2, for visualizing phylogenetic trees. (**A**) tree based on ITS sequence and inormative indels and (**B**) tree based on combined ITS sequence plus indels and morphlogical variation.

Figure 2 illustrates two phylogenetic trees of the species of the section *Trifolium* constructed using TreeGraph 2 [34] and generated by MrBayes, one based on analysis of ITS sequence and informative indels (Figure 2A) and the other on ITS sequence plus indels and morphlogical variation combined (Figure 2B); bootstrap values are represented on the branches. Similar species delimation was recorded in both trees that largely agree with that in Figure 1 except for grouping clades III and IV in a single clade in the tree based on both ITS sequence data and morphlogical variation. In both trees, clade I comprises 26 species and clade II 28 species. Clade I is divided into two major subclades based on the analysis of ITS data (Figure 2A), one comprises the same seven species isolated together and the other the same 19 species, differentiated as in Figure 1. Clade I in the tree based on the combined ITS and morphological data analysis (Figure 2B) comprises the same 26 species and most of the subclades are as recognized in Figure 1 and Figure 2A too. However, in Figure 2B, the following species are recognized as single branches: *T. dichroanthum*, *T. vavilovii* and *T. scutatum*, while *T. pallidum* and *T. pannonicum* form a small subclade. Of the remaining species, *T. alexandrinum*, *T. echinatum* and *T. clypeatum* are clustered from the other eighteen species, which are differentiated as one large subclade of seven species (*T. angustifolium* 1 and 2, *T. purpureum*, *T. leucanthum*, *T. palaestinum*, *T. apertum* and *T. canscens*) and a subclade of the four species *T. berytheum*, *T. carmeli* 1 and 2, and *T. meironense*). The remaining seven species are differentiated as *T. caucasicum*, one subclade of *T. plebeium*, *T. dasyrum* and *T. constantinopolitanum* and another of *T. miegeanum*, *T. obscurum* and *T. squarrosum.* The clustering of these species is congruent with their clustering in Figure 1.

In clade II, *T. latinum* and *T. ligusticum* are delimited as a small subclade from the other species in both Figure 2A,B. The four species *T. affine*, *T. arvense*, *T. bocconei*, *and T. dalmaticum* form a separate subclade in Figure 2A based on ITS data analysis but form a subclade grouped with a second subclade of seven species in the tree based on the analysis of ITS and morphological data combined (Figure 2B). These species are *T. incarnatum*, *T. leucanthum*, *T. stellatum*, *T. striatum*, *T. wettsteinii*, in addition to *T. molinerii* and *T. phleoides*. The same species are recognized as three small clades in Figure 2A. A small subclade of *T.* *ochroleucum* and *T. pratense* is recognized in both Figure 2A,B. The remaining species are delimited as three subclades in both Figure 2A,B: one of *T. lappaceum* 1 and 2 and a second of the five species *T. alpestre*, *T. medium*, *T. caudatum*, *T. diffusum* and *T. rubins*, and the third of six species (Figure 2A,B). On the other hand, clade III of four species and clade IV of two species are clearly isolated in the ITS-based tree (Figure 2A). These clades are grouped into clade III in the tree based on the combined analysis of ITS data and morphological features (Figure 2B).

## 3. Discussion

In the current study, the analysis of ITS data alone and in combination with variation in morphological traits produced different delimitation of species in the section *Trifolium* compared with the previous subsectional assignment of species as proposed by Zohary and Heller [14] who divided the section *Trifolium* into 17 subsections (Table 1). The analysis of the ITS data delimited the species of the section *Trifolium* in four clades, of which clade I of 26 species is divided into two major subclades, one including seven species (*T. alexandrinum*, *T. echinatum*, *T. scutatum*, *T. vavilovii*, *T. clypeatum*, *T. pallidum* and *T. pannonicum*). The clustering of these species, except *T. pallidum* and *T. pannonicum*, is congruent with views by a number of authors that represent the genetic resources of the cultivated species *T. alexandrinum*, known as Egyptian clover (Berseem). Aaronsohn [35] suggested *T. echinatum* M. B. from Palestine. Bobrov [36] claimed that *T. apertum* is the progenitor of *T. alexandrinum* based on morphological similarities. Putiyevsky et al. [8] considered other species related to *T. alexandrinum* such as *T. vavilovii*, *T. apertum*, *T. salmoneum*, *T. meironense* and *T. berytheum*. On the other hand, Trabut [37] and Eig [38] proposed *T. berytheum* from the coastal plains of Lebanon as the ancestor of *T. alexandrinum*. More recently, AFLP data analysis supported a close relationship of *T. alexandrinum* accessions from Syria and Egypt to *T. apertum*, *T. berytheum* and *T. salmoneum* as well as *T. clypeatum*, *T. plebeium*, *T. echinatum*, *T. constantinopolitanum* and *T. meironense* [18].

Ellison et al. [1] also reported the clustering of *T. alexandrinum*, *T. apertum* and *T. berytheum* as well as *T. scutatum*, *T. plebeium*, *T. vavilovii*, *T. echinatum* and *T. salmonium* in addition to *T. constantinopolitanum*. The grouping of *T. plebeium*, *T. berytheum* and *T. apertum* with *T. alexandrinum* is not well supported in the current results of the section *Trifolium* phylogeny. In the work of Zohary and Heller [14], *T. alexandrinum*, is placed in subsection Alexandrina with other four species, i.e., *T. apertum*, *T. berytheum*, *T. meironense* and *T. vavilovii*, whereas *T. plebeium* is placed in subsect. Clypeata with *T. clypeatum* and *T. scutatum*. In the meantime, *T. pallidum* is placed in the subsection Trifolium with *T. diffusum* and *T. pratense*, whereas *T. pannonicum* is placed with other six species in subsection Ochroleuca; these species are *T. canescens*, *T. caucasicum*, *T. caudatum*, *T. longidentatum*, *T.* *ochroleucum* and *T. trichocephalum*. However, in the current study, only *T. canescens*, *T. caucasicum* are in clade I while the others are in clade II. The delimitation of these species is generally congruent with their classification in Ellison et al. [1].

The other 19 species of clade I are delimited as a main subclade of 12 species and two small subclades, one of *T. berytheum*, *T. carmeli* 1 and 2 and *T. meironense*, and the other of *T. miegeanum*, *T. obscurum* and *T. squarrosum*. The 12 species in this subclade are differentiated as single branches (Figure 1) and are divided in different subsections by Zohary and Heller [14]; *T. berytheum* and *T. meironense* belong to one subsect. Alexandrina and *T. carmeli* belongs to subsection Echinata. On the other hand, *T.* *miegeanum*, *T. obscurum* and *T.* *squarrosum* belong to subsection Urceolata. Another two species of the same subsection are separated in clade II; these are *T. constantinopolitanum* and *T. leucanthum*.

The clade II of 28 species is differentiated in small subclades based on the ITS data analysis including one clade of four species (*T. affine*, *T. arvense*, *T. bocconei* and *T. dalmaticum*). The same subclade is also differentiated in the other trees based on the analysis of informative indels of ITS sequence alone or in combination with the morphological data. The delimitation of *T. affine*, *T. arvense*, *T. bocconei* agrees with the results of Ellison et al. [1] but only *T. affine*, *T. arvense* are placed in subsect, Arvensia by Zohary and Heller [14], *T. bocconei* was placed in subsection Trichoptera with *T. trichopterum* and *T. dalmaticum* was placed in subsection Scabroidea with *T. lucanicum* and *T. scabrum*. In the study of Ellison et al. [1], *T. dalmaticum* was clustered with *T. scabrum* and *T. lucanicum*.

Based on ITS analysis (Figure 1 and Figure 2A), clade III comprises *T. hirtum*, *T. trichopterum*, *T. haussknechtii* and *T. sylvaticum*, and clade IV comprises *T. cherleri* and *T. scabrum.* Combining ITS data with morphological characters, both clades appeared as two subclades of clade III (Figure 2B). In Ellison et al. [1], *T. trichopterum* was also clustered with *T. haussknechtii*, and *T. sylvaticum*, while *T. hirtum* was clustered with *T. cherleri*. The clustering of these specie differs with their distribution in the subsections proposed by Zohary and Heller [14], who placed *T. cherleri*, *T. hirtum* and *T. lappaceum* in subsection Lappacea and *T. haussknechtii* with *T. angustifolium. T. dasyurum*, *T. dichroanthum* and *T. palaestinum* in subsection Angustifolia and grouped *T. sylvaticum* with *T. incarnatum*, *T. molinerii* and *T. stellatum* in subsection Stellata.

The classification of the species in the section *Trifolium* in three clades, clade I of 26 species, clade II of 28 species and a small clade III of six species in Figure 2B, is generally comparable to their delimitation by Ellison et al. [1], although these authors regarded the species of the section *Trifolium* as two clades, A and B. Clade A (25 species) generally corresponds to clade I of the present study, whereas clade B generally corresponds to clade II and clade III of this study. Ellison et al. [1] assumed that interspecific relationships are better resolved within clade B than within clade A. However, the species branching in clade B of Ellison et al. [1] indicate a clade of *T. haussknechtii*, *T. sylvaticum* and *T. trichopterum*, associated with *T.* *cherleri* and *T. hirtum*, representing five of the six species differentiated as clade III in the tree constructed using ITS sequence and informative indels in combination with morphological data (Figure 2B).

As circumscribed by Zohary and Heller [14], the section *Trifolium* is primarily defined by sessile and ebracteate flowers, a hairy or callous ring or a bilabiate protrusion at the throat of the calyx tube, a calyx limb with unequal teeth, a one-seeded pod enclosed in the calyx tube that lacks sutures and ruptures transversely at maturity. The annual life history is the result of some type of time-limitation supporting the shortened life cycle, which is endorsed by inbreeding (Snell and Aarssen [39]. Inbreeding in annuals has evolved as a result of strong re-selection, leading to either selection for a shorter time to complete the reproductive cycle, or selection for shorter pollination time [40]. The habit form affects the level of genetic diversity in *Trifolium* as the intraspecific genetic variation in annuals is significantly lower than in perennials [41]. Most of the morphological characters give no clear boundaries in the sub-sectional classification of the section *Trifolium*. Most of the flowering heads are ovoid in the terminal position, with a few species having mixed, terminal and axil flowering heads. Additionally, most of the species has solitary flowering heads on each branch. As for the flower, the corolla is slightly larger than calyx or about twice its length; however, in some species, corolla is equal to the calyx. Most of the species have sharp calyx teeth. The calyx is mostly 10-nerved but few species have 15–20-nerved calyxes; however, the distribution of calyx nerves is not associated with the sub-sectional delimitation of the species, as proposed by Zohary and Heller [14], or their delimitation based on the classification of species based on molecular data [1,16]. Meanwhile, the use of morphological traits in the constructions of phylogenetic relationships of the species in the section *Trifolium* led to stabilization of the tree topology when combined with the ITS data as shown by Figure 2B.

The section *Trifolium* has been shown to be heterogeneous in seed proteins [17], and chromosome numbers of 2n = 16, 14, 12 or 10 have been reported for the species in the genus [14,17,23,24,26]. It is, however, evident that the vast majority of species in clade I have 2n = 16, whereas a diploid number of 2n = 14 was only recorded in *T. purpureum*; meanwhile, 2n = 16 and 2n = 32 were recorded for *T. angustifolium.* On the other hand, in clade II, 14 of the 28 species of clade II have 2n = 10, 2n = 12 and 2n = 14 and seven species have only 2n = 16. A diploid number of 2n = 14 was recorded in twelve species of clade II, but in three of them 2n = 16 was also recorded. The seven species in which only 2n = 14 was recorded are *T. molinerii*, *T. phleoides*, *T. striatum*, *T. gemellum*, *T. stellatum*, *T. incarnatum* and *T. arvense*. In *T. dalmaticum*, 2n = 10 was scored and in *T. affine*, 2n = 12 and 2n = 16 were also scored. Of the six species delimited as clade III, 2n = 10 was scored in *T. cherleri*, *T. hirtum* and *T. scabrum*, and 2n = 14 was scored in *T. sylvaticum* and *T. trichopterum.* Polyploidy based on *x* = 8 has been reported in only three species, i.e., *T. medium*, T. *pannonicum* and *T. pratense*. Integration of linkage and chromosome maps for the latter species demonstrated chromosomal collinearity among allogamous varieties and should provide valuable insight into allogamous legume genetics [46].

The majority of *Trifolium* species have a basic chromosome number of *x* = 8, which is regarded as the primitive basic number of the genus [14,17,25]. Assuming that *x* = 8 is the basic number for the genus *Trifolium*, from which *x* = 7, 6 and 5 were derived through successive aneuploidy events, species in clade III and clade II may be regarded as more evolved than species in clade I. In the genus as a whole, species with *x* = 8 are found in all eight sections of Zohary and Heller [14], while *x* = 7 is confined to section *Chronosemium*, section *Trifolium* and section *Trichocephalum*, and *x* = 6 and 5 to the latter two sections only. These two sections may be regarded the most advanced sections of the genus. However, the conflicting reports of different chromosome numbers for the same species as given in Table 1 make it difficult to conclude phylogenetic relationships based on chromosome number variations from the published literature. Moreover, careful identification of material used in addressing systematics and phylogeny of species in *Trifolium* must be thoughtfully checked by careful morphological description.

In conclusion, the grouping of species as reported here and by Ellison et al. [1] does not support the delimitation of species in the section *Trifolium* in 17 subsections as described by Zohary and Heller [14]. The species of subsections *Alexandrina* and *Clypeata*, in addition to most of the species of subsections Angustifolia, Echinata and Urceolata and half of the species of subsection Ochroleuca, are grouped in clade I of the present study. Additionally, the species of subsections Alpesteria, Intermedia, Phleoidea, Arvensia and Stenosemium (*T. striatum*) are grouped together in clade II. The remaining species are distributed in the three clades. In the genus *Trifolium*, the basic number of *x* = 8 is the number from which *x* = 7, 6 and 5 were derived through successive aneuploidy events. With reference to the distribution of these numbers in the section *Trifolium*, species in clade III and clade II appear to be more evolved than species in clade I.

## 4. Material and Methods

### 4.1. Plant Material

Seed material of 60 of the 73 species of the section *Trifolium* was obtained from a variety of sources (Table 2). Seeds were germinated and grown to maturity at the Botanic Garden of Miami University, Oxford Ohio, USA, to confirm their taxonomic status. Voucher specimens of all species are deposited at the Willard Sherman Turrell Herbarium of Miami University. Ten morphological traits were used as diagnostic characters with reference to their description by Zohary and Heller [14].

### 4.2. DNA Isolation and DNA Sequence

For ITS sequencing, total genomic DNA was isolated from fresh leaves of seedlings using the 2X CTAB procedure [47]. A few leaflets were powdered, in liquid nitrogen, using mortar and pestle, and homogenized in 0.75 mL of hot 4× CTAB buffer, to which 1% PVP, 1% Na-bisulphite and 0.2% β-mercaptoethanol were added before use, and transferred to 2.0 mL microfuge. The tubes were incubated for 30 min at 60 °C in a water bath with occasional gentle mixing. After incubation, the mixture was emulsified with 0.5 mL of chloroform-isoamyl alcohol (24:1) and centrifuged at 10,000 g for 5 min. The aqueous top layer was pipetted into a new tube, mixed with 0.5 mL cold isopropanol, kept at −20 °C for 30 min and centrifuged at 12,000 g for 10 min. The alcohol was discarded, and the pellet was washed in 0.75 mL 76% EtOH/0.01 M NH_4_OAC for 5 min followed by washing in 0.75 mL 76% EtOH/0.01 M NaOAC for a few minutes. The pellet was then dried and suspended in 0.2 mL TE buffer, 1 µL RNase was added to remove RNA and the buffer extract was kept at 37 °C for 30 min before using DNA for ITS amplification.

The ITS region was amplified using primers of White et al. [48]. The spacers were sequenced on an ABI PRISM 310^®^ Genetic Analyzer using capillary sequencing and Big Dye Terminator Chemistry (Applied Biosystems, Inc., Foster City, CA, USA). The sequence boundaries of the spacers were determined by comparison to published *Trifolium* sequences [1,16] and complete sequences were deposited in GenBank (Table 2). All ITS sequences were aligned using Clustal [49], with manual gap adjustments made to improve the alignment. Indels were coded following Simmons and Ochoterena [50] using the Gap Coder software [51].

### 4.3. Phylogenetic Analysis

Two approaches were used to express the phylogenetic relationships of the examined species based on the molecular ITS sequencing alone and in combination with the morphological characters coded as multistate matrix (supplementary Table S1). The Bayesian analysis expressing the cladogenesis of species was expressed as cluster using the software MrBayes 3.2 [32]. The best-fit substitution model (SYM + G) was chosen based on the Akaike Information criterion (AIC) as determined by the MrModel-test v.2.3 [52]. The Markov chain Monte Carlo (MCMC) process was run for 3,000,000 generations and trees were sampled every 1000 generations with 16 chains. Stationarity was accomplished when “the average standard deviation of split frequencies” remained  <0.01; the first 25% of runs were discarded. The phylogenetic tree established with MrBayes 3.2 was represented with Interactive Tree of Life (iTOL) tools [33] and TreeGraph 2 software [34].

## Figures and Tables

**Table 1:** Section *Trifolium* species chromosome numbers and proposed sub-sectional delimitation as proposed by Zohary and Heller [14] and the corresponding classification as three clades in the current study. The listed chromosome numbers searched in the Chromosome Counts Database: http://ccdb.tau.ac.il/Angiosperms/Leguminosae/Trifolium/ and the Missouri Botanical Garden (accessed on 15 June 2021). http://legacy.tropicos.org/Name/40018244?projectid=9 (accessed on 15 June 2021). The 2n cromosomes counts for the following five species were taken from the refrence for each species as follows: *T. apertum* [42] *T. dasyurum* [43], *T. dichroanthum* [14], *T. carmeli* [44], *T. trichocephalum* [14], *T. haussknechtii* [45].

*Trifolium* Species	2n Chromosome Number	Subsection	New Clade
*T. alexandrinum*	16	Alexandrina	Clade I
*T. vavilovii*	16		
*T. apertum*	16		
*T. berytheum*	16		
*T. meironense*	16		
*T. scutatum*	10, 16	Clypeata	
*T. clypeatum*	16		
*T. plebeium*	16		
*T. pallidum*	16	Trifolium	
*T. lucanicum*	-	Scabroidea	
*T. dasyurum*	16	Angustifolia	
*T. dichroanthum*	16		
*T. palaestinum*	16		
*T. angustifolium*	14, 16, 32		
*T. purpureum*	14, 16		
*T. carmeli*	14	Echinata	
*T. echinatum*	16		
*T. canescens*	-	Ochroleuca	
*T. caucasicum*	-		
*T. pannonicum*	64, 96, 98~180		
*T. miegeanum*	16	Urceolata	
*T. obscurum*	16		
*T. squarrosum*	16		
*T. constantinopolitanum*	16		
*T. striatum*	14	Stenosemium	Clad II
*T. latinum*	16	Echinata	
*T. alpestre*	16	Alpesteria	
*T. rubens*	16		
*T. medium*	48, 64, 78, 80, 84, 96, 98, 126	Intermedia	
*T. heldreichianum*	16		
*T. patulum*	-		
*T. velebiticum*	-		
*T. wettsteinii*	-		
*T. caudatum*	-	Ochroleuca	
*T. longidentatum*	-		
*T. trichocephalum*	14, 28		
*T. ochroleucum*	16		
*T. gemellum*	14	Pheloidea	
*T. phleoides*	14		
*T. ligusticum*	12		
*T. leucanthum*	14, 16	Urceolata	
*T. diffusum*	16	Trifolium	
*T. pratense*	14, 16, 28, 56		
*T. molinerii*	14	Stellata	
*T. incarnatum*	14		
*T. stellatum*	14		
*T. lappaceum*	16	Lappacea	
*T. affine*	12, 14, 16	Arvensia	
*T. arvense*	14		
*T. bocconei*	12, 14	Trichoptera	
*T. dalmaticum*	10	Scabroidea	
*T. sylvaticum*	14, 16	Stellata	Clade III
*T. hirtum*	10	Lappacea	
*T. cherleri*	10		
*T. haussknechtii*	16	Angustifolia	
*T. scabrum*	10, 16	Scabroidea	
*T. trichopterum*	14	Trichoptera	

**Table 2:** *Trifolium* species names, source and accession number *, country of origin and Gene Bank submission number for ITS1/ITS2 sequences.

No	*Trifolium* Species	Source and Accession Number *	Country of Origin	Gene Bank ITS1/ITS2
1	*T. affine* Presl.	SRPIS 369014	Turkey	MW683982/MW684022
2	*T. alexandrinum* L.	ARC, EG 10	Egypt	AF154381/AF154605
3	*T. alpestre* L.	Kew 11097	Switzerland	AF154400/AF154624
4	*T. angustifolium* L.	MU 9491	USA	MW683983/MW684023
5	*T. angustifolium* L.	BGUZ 8023	Switzerland	AF154388/AF154612
6	*T. apertum* Bobrov	SRPIS 314117	Russia	MW683984/MW684024
7	*T. arvense* L.	SRPIS 120077	Turkey	MW683985/MW684025
8	*T. berytheum* Bois. and T. Blanche	SRPIS 369019	Turkey	MW683986/MW684026
9	*T. bocconei* Savi	SRPIS 224643	Morocco	MW683987/MW684027
10	*T. canescens* Willd.	WRPIS 418866	Italy	MW683988/MW684028
11	*T. carmeli* Boiss.	IPK TRIF 100/75	USA	MW683989/MW684029
12	*T. carmeli*	SRPIS 353422	Israel	MW683990/MW684030
13	*T. caucasicum* Tausch*	WRPIS 597496	Russia	MW683991/MW684031
14	*T. caudatum* Boiss.	WRPIS 251858	Italy	MW683992/MW684032
15	*T. cherleri* L.	Italy 589	Italy	AF154357/AF154582
16	*T. clypeatum* L.	ICARDA 1149	Syria	AF154383/AF154607
17	*T. constantinopolitanum* Sert.	ICARDA 1114	Syria	AF154386/AF154610
18	*T. dalmaticum* Vis.	WRPIS 516292	Serbia	MW683993/MW684033
19	*T. dasyurum* C. Presl.	SRPIS 369029	Australia	AF154387/AF154611
20	*T. dichroanthum* Boiss.	SRPIS 292474	Israel	MW683994/MW684034
21	*T. diffusum* Ehrh.	Kew 1094	Serbia	AF154401/AF154625
22	*T. echinatum* N.B.	ICARDA 1758	Syria	AF154382/AF154606
23	*T. gemellum* Willd.	SRPIS 302967	Spain	MW683995/MW684035
24	*T. haussknechtii* Boiss.	WRPIS G31176-98i	Bulgaria	MW683996/MW684036
25	*T. heldreichianum* Hausskn.	WRPIS 419289	Greece	MW683997/MW684037
26	*T. hirtum* All.	Kew 25085	Greece	AF154359/AF154932
27	*T. incarnatum* L.	Kew 55767	England	AF154392/AF154932
28	*T. lappaceum* L.	ICARDA 108405	Syria	MW683998/MW684038
29	*T. lappaceum* L..	Italy 529	Italy	AF154395/AF154619
30	*T. latinum* Sebast.	IOWA State	USA	MW683999/MW684039
31	*T. leucanthum* M.B.	ICARDA 1111	Syria	AF154394/AF154618
32	*T. ligusticum* Lois.	SRPIS 419415	Greece	MW684000/MW684040
33	*T. longidentatum* Nabelek	WRPIS 542831	Bosnia, Herzegovina	MW684001/MW684041
34	*T. lucanicum* Gasp. Ex Guss.	Kew 13921	Greece	AF154390/AF154614
35	*T. medium.* L.	WRPIS 325481	Russia	MW684002/MW684042
36	*T. meironense* Zoh. and Lern.	ICARDA 69062	Algeria	MW684003/MW684043
37	*T. miegeanum* Maire	SRPIS 258406	Portugal	MW684004/MW684044
38	*T.molinerii* (*incarnatum* L.)	SRPIS 591667	Bulgaria	MW684005/MW684045
39	*T. obscurum* Savi	SRPIS 369057	Italy	MW684006/MW684046
40	*T. ochroleucum* Huds.	Kew 7559	Greece	AF154397/AF154621
41	*T. palaestinum* Boiss.	SRPIS 369060	Palestine	MW684007/MW684047
42	*T. pallidum* Waldst. and Kit.	Kew 31516	Greece	AF154385/AF154609
43	*T. pannonicum* Jacq.	BGUZ s.n.	Switzerland	AF154384/AF154608
44	*T. patulum* Tausch	WRPIS 604678-97i	Serbia	MW684008/MW684048
45	*T. phleoides* Willd.	SRPIS 298420	Turkey	MW684009/MW684049
46	*T. plebeium* Boiss.	ICARDA 67905	Syria	MW684010/MW684050
47	*T. pratense* L.	JBVU 1044	France	AF154396/AF154620
48	*T*. *purpureum* Loisel	ICARDA 938	Syria	AF154389/AF154613
49	*T. rubens* L.	IPP 135	France	AF154398/AF154622
50	*T. scabrum* L.	ICARDA 939	Syria	AF154358/AF154583
51	*T. scutatum* Boiss.	ICARDA 67459	Syria	MW684011/MW684051
52	*T. squarrosum* L.	SRPIS 233720	Italy	MW684012/MW684052
53	*T. stellatum* L.	ICARDA 947	Syria	AF154393/AF154617
54	*T. striatum* L.	Kew 5337	England	AF154391/AF154615
55	*T. sylvaticum* Gerard	SRPIS 120194	Turkey	MW684013/MW684053
56	*T. trichocephalum* Bieb.	WRPIS 251210-64	North Macedonia	MW684014/MW684054
57	*T. trichopterum* Pancic	SRPIS 583430	Bulgaria	MW684015/MW684055
58	*T. vavilovii* Eig	Vencent 11160	USA	MW684016/MW684056/
59	*T. velebiticum* Degen	WRPIS G30679-97i	USA	MW684017/MW684057
60	*T. wettsteinii* Dorfl. and Hay	WRPIS G31121	USA	MW684018/MW684058

ARCE = Agriculture Research Center, Cairo, Egypt. BGUZ = Botanischer Gaten der Universität Zurich, Switzerland. ICARDA = International Center for Agricultural Research in Dry Areas, Aleppo, Syria. ICLA = International Livestock Center for Africa, Addis Ababa, Ethiopia. IPP = University of Bayreuth Botanic Garden, Germany. Italy = Universita di Siena, Italy. JBVU = Jardin Botanique de la Ville et de I’Universite, France. Kew = Royal Botanic Garden, Kew, Richmond, Surry, London. UK. MU = Miami University, Oxford Ohio, USA. SRPIS = South Regional Plant Introduction Station, USA. WRPIS = South Regional Plant Introduction Station, USA.

## Data Availability

Date is contained within the article.

## Supplementary Materials

The following are available online at https://www.mdpi.com/article/10.3390/plants10101985/s1, Table S1: Morphological characters of the examined *Trifolium* species and their recorded 2n chromosome numbers.
