# Supplementary material for: Molecular Phylogeny of Trifolium L. Section Trifolium with Reference to Chromosome Number and Subsections Delimitation
Source: Plants (Basel). 2021 Sep 23;10(10):1985. doi: 10.3390/plants10101985 (PMC8539877; doi:10.3390/plants10101985)
Supplement: Supplementary file 1 [file plants-10-01985-s001.zip › plants-1312975-supplementary.pdf]

SUPPLEMENTARY MATERIALS

Molecular Phylogeny of *Trifolium* L. Section *Trifolium* with Reference to Chromosome Number and Subsections Delimitation

Hanan I. Sayed Ahmed[1], Abdelfattah Badr[2], Hanaa H. El-Shazly[3], Linda Watson[4], Ahmed S. Fuoad[5*] and Faten Y. Ellmouni[6]

Supplementary Table S1. Morphological characters of the examined *Trifolium* species and their recorded 2n chromosome numbers

	***Trifolium* species**	**Life form**	**No. of heads/ branch**	**Fl head position**	**Fl head shape**	**Fl head size**	**Calyx teeth**	**No. of calyx nerves**	**Corolla color**	**Corolla /calyx**	**2n**
1	***affine***	Annual	Twin, solitary	Terminal	Ovoid cylindrical	1-3	Sharp	10	White, pink	Equal	12, 14, 16
2	***alexandrinum***	Annual	Solitary	Terminal, axile	Ovoid	1.5 - 2		10	Cream	Longer	16
3	***alpestre***	perennial	Solitary, terminal	Terminal	Globular ovate	2	Blunt	15-20	Purple	Longer	16
4	***angustifolium***	annual	Solitary	Terminal	Cylindrical	3-8	Sharp	-	Pink, purple w plots	Equal	14, 16, 32
5	***apertum***	annual	Solitary	Terminal, axile	Rhomboid	1.5 – 12.5	-	10	Yellow, cream	Longer	16
6	***arvense***	annual	Solitary	Axile, terminal	Ovoid	1 - 2	-	-	white, pink	Equal	14
7	***berytheum***	annual	Solitary	terminal, axile	Obconical	1-2	-	10	Yellow, cream	Longer	16
8	***bocconei***	annual	Solitary, twins	Terminal, axile	Ovoid	1-2		10	Pink, Reddish	Equal	12, 14
9	***canescens***	perennial	Solitary	Terminal	Ovoid	2-4	Sharp	10	yellow	Twice	-
10	***carmeli***	annual	Solitary	Terminal		2-6	Sharp	10	Red, pink	Longer	14
11	***caucasicum***	perennial	Solitary	Terminal	Ovoid	-	Sharp	10	cream	Longer	-
12	***caudatum***	perennial	Solitary	Terminal	Ovoid	2-2.5	Sharp	10	Pink, purple	Twice	-
13	***cherleri***	Annual	Solitary	terminal	Ovoid Globular	0.6-1.2	Blunt	20	White, cream	Equal	10
14	***clypeatum***	Annual	Many		Obovoid	2-3	Sharp	-	Pink, whitish	Twice	16
15	***constantinopolitanum***	Annual	Solitary	Terminal	Ovoid obconical	1.2-2.2	-	10	Pink, white	Equal	16
16	***dalmaticum***	Annual	Solitary	Terminal	Elongate	1-2	-	10	Pink, purple	Twice	10
17	***dasyurum***	Annual	Solitary	Terminal	Ovoid	1.5-3	-	-	purple	Equal	16
18	***dichroanthum***	Annual	Solitary	Terminal	Ovoid	2-6	Blunt	-	Two colors	Longer	16
19	***diffusum***	Annual	Solitary	Terminal	Globular	~3	Blunt	10	Purplish pink	Equal	16
20	***echinatum***	Annual	Solitary	Terminal, Axile	Ovoid	1.2-3	-	10	Cream		16
21	***gemellum***	Annual	Twin	Terminal	Ovoid	0.8-1.8	-	-	pink	Equal	14

22	***hausskn*echtii**	Annual	Solitary	Terminal, axile	Ovoid	1.5-1.8	blunt	10	White, cream	Equal	16
23	***heldreichianum***	Perennial	Twin	terminal	Globular	2	Sharp	10	Pink	Twice	16
24	***hirtum***	Annual	Solitary	terminal	Globular	2	Blunt	10	Purple	Longer	10
25	***incarnatum***	Annual	Solitary	terminal	Oblong	2-6	Sharp	10	Red, pink	Longer	14
26	***lappaceum***	Annual	Solitary	Terminal, Axile	Globular	1-1.4		15-20	White, pink	Equal	16
27	***latinum***	Annual		Terminal		1.5-2		10	White, pink, with blots	Twice	16
28	***leucanthum***	Annual	Solitary	Terminal	Globular	1-1.5		10	Cream, pink with blots	Equal	14, 16
29	***ligusticum***	Annual	Solitary	Terminal, axile	Ellipsoidal, ovoid	1-2	-	10	Purple, pink	Equal	12
30	***longidentatum***	Perennial	Solitary	Terminal	Ovoid	2-4	Sharp	-	Pale pink	Twice	-
31	***lucanicum***	Annual	Twin	Axile, terminal	Elongate	1-2	Blunt	-	Flesh	Longer	-
32	***medium***	Perennial	Solitary	Terminal,	Globular, ovoid	1.5-2	Sharp	12-20	purple	Twice	48, 64, 78, 80, 84, 96, 98, 126
33	***meironense***	Annual	Solitary, pairs	Terminal axile	Ovoid oblonge	1-1.5		10	Purple, pink	Longer	16
34	***miegeanum***	Annual	Solitary	Terminal, axile	Ovoid	2- 3.5	Sharp	10	White	Equal	16
35	***molinerii***	Annual	Solitary	Terminal		2-6	Sharp	10	Red, pink	Longer	14
36	***obscurum***	Annual	Solitary	Terminal	Globular ovoid	1.5-2	Sharp	10	Whitish, pink	Equal	16
37	***ochroleucum***	Perennial	Solitary	Terminal	Globular, ovoid	1-2	Sharp	-	Cream	Twice	16
38	***palaestinum***	Annual	Twin, Solitary	Terminal	Ovoid	1.5-2	-	-	White	longer	16
39	***pallidum***	Annual	Solitary	Terminal	Globular, ovoid	1.5-2	Blunt	10	White, cream	Twice	16
40	***pannonicum***	Perennial	Solitary	Terminal	Ovoid	2-4	Sharp	10	purple	Twice	64, 96, 98~180
41	***patulum***	Perennial	Twin	Terminal	Ovoid	2-3	Blunt	10	purple	Longer	-
42	***phleoides***	Annual	solitary	Terminal	Ovoid	1-2		10	Purple, pink	Equal	14
43	***plebeium***	Annual		Terminal	Ovoid	1-2	-	10	White cream	Twice	16
45	***pratense***	Perennial	solitary	Terminal	Globular, Ovoid	1.5-1.8	Blunt	10	Redish purple, pink	Twice	14, 16, 28, 56
46	***purpureum***	Annual	Many	Terminal	Ovoid oblong	~2	-	-	Purple, lilac	Longer	14, 16

47	***rubens***	Perennial	Twin	Terminal, axile	Oblong Cylendrical	3-7	Blunt	15-20	purple	Longer	16
48	***scabrum***	Annual	Twin	Axile	Ovoid	0.7-1			White, pink	Equal	10, 16
49	***scutatum***	Annual		Terminal	Ovoid	1.8-2		10	White, cream with blots	Twice	10, 16
50	***squarrosum***	Annual	Solitary	Terminal	Globular Ovoid	~2.5	Sharp	10	Whitish pink	Equal	16
51	***stellatum***	Annual	Solitary	Terminal, axile	Globular, Ovoid	1.5-2	Sharp	10	Pink, white	Equal	14
52	***striatum***	Annual	Solitary, Pairs	Terminal, axile	Ovoid	0.6-1.5	Sharp	10	pink	Equal	14
53	***sylvaticum***	Annual	Solitary, twin	Terminal	Globular ovoid	1-1.5	-	10	Pink, purple	Longer	14, 16
54	***trichocephalum***	Perennial		Terminal	Ovoid	4-6	blunt	10	Cream	Twice	14, 28
55	***trichopterum***	Annual	Solitary, Twin	Axile, terminal	Globular, Ovoid	1-2.5	Sharp	10	Cream	Longer	14
56	***vavilovii***	Annual	Solitary	Terminal	Obconical, Ovoid	2-2.5	-	10	White, Ccream	Longer	16
57	***velebiticum***	Perennial	Solitary	Terminal	Globular				Pink	Longer	-
58	***wettsteinii***	Perennial	Solitary	Terminal	Obvoid	2	Sharp	10	Pink	Twice	-
